# The Costs of Concern: Health Implications of Worries about Aging Parents and Adult Children

**DOI:** 10.1093/geroni/igab046.2914

**Published:** 2021-12-17

**Authors:** Kelly Cichy, Athena Koumoutzis

**Affiliations:** 1 Kent State University, Kent, Ohio, United States; 2 Miami University, Oxford, Ohio, United States

## Abstract

As their parents age and their children enter adulthood, midlife adults need to manage their worries and concerns about both generations. In midlife, worries about aging parents’ health and emerging needs for support co-occur alongside worries about adult children’s relationships and prolonged need for support. Research reveals links between midlife adults’ worry and sleep quality, underscoring how worries compromise health and well-being. In addition to compromising sleep, worries may also contribute to poor health behaviors, such as emotional eating. Emotional eating, where individuals eat in response to stressors and negative emotions, is a significant risk factor for overeating and obesity. Less is known; however, about how midlife adults’ worries contribute to poor health behaviors. To address this gap, the current study considers how midlife adults’ concurrent and previous day’s daily worries about aging parents and adult children are associated with daily well-being and health behaviors. Respondents are midlife adults (40-60 years) from Wave II of the Family Exchanges Study (Fingerman et al., 2009). During 7 days of daily telephone interviews, respondents indicated if they worried about their adult children and their aging parent(s), if they ate food for comfort, and their daily negative mood. Controlling for demographics, on days when midlife adults worried about their adult child(ren), they reported more negative emotions than on days without these worries (p <.05). Respondents engaged in more eating for comfort the day after they reported worrying about their mother (p < .05). Implications for aging families will be discussed.

